# Many ways, one microorganism: Several approaches to study *Malassezia* in interactions with model hosts

**DOI:** 10.1371/journal.ppat.1010784

**Published:** 2022-09-08

**Authors:** Kevin Ehemann, María Juliana Mantilla, Felipe Mora-Restrepo, Andrea Rios-Navarro, Maritza Torres, Adriana Marcela Celis Ramírez

**Affiliations:** Grupo de Investigación Celular y Molecular de Microorganismos Patógenos (CeMoP), Departamento de Ciencias Biológicas, Universidad de los Andes, Bogotá, Colombia; McGill University, CANADA

## Abstract

*Malassezia*, a lipophilic and lipid-dependent yeast, is a microorganism of current interest to mycobiologists because of its role as a commensal or pathogen in health conditions such as dermatological diseases, fungemia, and, as discovered recently, cancer and certain neurological disorders. Various novel approaches in the study of *Malassezia* have led to increased knowledge of the cellular and molecular mechanisms of this yeast. However, additional efforts are needed for more comprehensive understanding of the behavior of *Malassezia* in interactions with the host. This article reviews advances useful in the experimental field for *Malassezia*.

## Introduction

The *Malassezia* genus was discovered in the early 19th century. Currently, 18 species belonging to this genus have been identified, all of which are considered to be important contributors to the human and animal mycobiota [1,2]. In addition to the 18 current species, the incorporation of *M*. *auris*, *M*. *palmae* and *M*. *rara* has been recently proposed [1]. *Malassezia* yeast can cause some dermatological conditions, such as pityriasis versicolor, atopic dermatitis, dandruff, seborrheic dermatitis, and folliculitis but also plays a role in systemic infections. Recently, *Malassezia* has been associated with chronic diseases such as inflammatory bowel disease (IBD), cancer, and neurological disorders such as Alzheimer’s disease [3]. These findings continue to attract attention and emphasize the importance of understanding the role of this microorganism in human health.

Experimental approaches allow the study of this yeast at the cellular and molecular level. In addition, genomic data from multiple species are now available, allowing the identification of *Malassezia* genes that are involved in environmental adaptation, metabolism, pathogenicity, and antifungal resistance [4]. The use of a convenient system of genetic manipulation through *Agrobacterium tumefaciens*–mediated transformation (AMT) and a CRISPR/Cas9 system could increase the ability to study gene function and provide information about pathogenicity [4].

Given its role in the human mycobiota, it is important to understand how *Malassezia* interacts with the host. Both in vitro (ex vivo) and in vivo models have been studied, as summarized in Table 1. An in vivo mammalian skin model using C57BL/6 mice, which exhibits similarities to the human immune system, facilitated the elucidation of a key role of *Malassezia* in inducing a Th17 response associated with exacerbation of skin inflammation [5]. In addition, a murine model has been used to study the role of *Malassezia* in IBD and the pathogenesis of pancreatic ductal adenocarcinoma (PDA). Interestingly, a mouse model led to the finding that *M*. *restricta* has a negative association with Huntington’s disease [5–8]. Unfortunately, a murine model also has disadvantages, including ethical concerns and high cost. However, invertebrate models, such as *Galleria mellonella*, may be alternatives, offering advantages such as easy management, low cost, and similar immune response to humans [9]. In addition, ex vivo models (for instance, skin explants) and in vitro models (for instance, keratinocyte cell lines and reconstructed human epidermis) provide conditions similar to the human skin to study the human immune response [10–12].

**Table 1:** Different studies applying experimental models to study *Malassezia*–host interactions.

Current models to study host–microbe interaction				Studies performed in *Malassezia* species		
Model category	Experimental model	Advantages	Disadvantages	*Malassezia* species	Methodological assessment/parameters analyzed	Findings
Invertebrates	*Galleria mellonella*	• Conserved innate immune system • Low cost • No ethical implications • Minimal infrastructure requirements • Easy management [9]	• No adaptive immune response • No fully sequenced genome [13,14]	*M*. *furfur* *M*. *pachydermatis*	• Histological stain: H&E • Confocal microscopy • Larval melanization and survival • Fungal burden • Hemocyte response	*G*. *mellonella* is a suitable model to evaluate the *Malassezia*–host interaction process, where the survival of larvae is dependent on inoculum concentration, species of *Malassezia*, and incubation temperature [9]
	*Drosophila melanogaster*	• Short life cycle • Low cost • Genome available • Available advanced technologies to apply • Conserved innate immune system [15]	• No adaptive immune response [13]	*M*. *pachydermatis*	• Histological stain: HE, GMS • Fly survival • Fungal burden • Systemic infection	Toll-deficient flies infected with *M*. *pachydermatis* are susceptible to infection, and they are inoculum dependent compared to wild type [16]
	*Caenorhabditis elegans*	• Short life cycle • Physiologically simple • Genome available • Transparent cuticle • Easy to obtain and manage [14]	• No adaptive immune response • Unable to be cultured at 37°C • Difficult to inoculate [14]	*M*. *pachydermatis*	• Worm survival	*C*. *elegans* showed a high mortality after 96 h of exposure to plates incubated with *M*. *pachydermatis* at 25°C [17]
Vertebrates	Mouse	• Conserved immune response • Various routes of fungal administration (intravenous, cutaneous, ocular, vaginal, intragastric, oropharyngeal) • Development of systemic symptoms of infection • Sequenced genome [13,18]	• High cost • Ethical implications • Special requirements (larger space, optimal asepsis) • Experience and training are needed [13]	*M*. *pachydermatis*	• Histological stain: HE, methenamine silver stain • Fungal burden	*M*. *pachydermatis* causes otitis and dermatitis in mice, with a high burden at the beginning of infection that decreases over time [19]
				*M*. *sympodialis*	• RT-qPCR • FISH	Coinfection of mice with *M*. *sympodialis* and *Pseudomonas aeruginosa* or *Staphylococcus aureus* influence the immune response of the host [20]
				*M*. *pachydermatis* *M*. *furfur* *M*. *sympodialis*	• RT-qPCR • Histochemistry • Immunofluorescence • Flow cytometry and cell sorting • Histological stain: HE, periodic acid-Schiff	*Malassezia*-induced IL-17 immune response in the skin results in fungal reduction and promotes inflammation [5].
				*M*. *restricta*	•Morphological evaluation • Histological stain: HE • Flow cytometry • qPCR	The presence of *M*. *restricta* did not affect the mouse colon but exacerbated DSS-induced colitis. *M*. *restricta* led to severe intestinal inflammation with higher production of IL-17A- and IFN-γ-producing CD4+ cells [6]
				*M*. *restricta*	• Shotgun metagenomic sequencing	*M*. *restricta* was a key species in the gut microbiome with a negative association with Huntington’s disease in the R6/1 transgenic mouse model [8]
				*Malassezia* spp. *M*. *globosa*	• qPCR • Histological stain: HE, Gomori trichrome • IHC and microscopy • FISH • DNA sequencing	*Malassezia* spp. can migrate from the gut to the pancreas, and its presence there is higher in mice with PDA. This may be mediated by activation of the MBL pathway [7]
				*Malassezia furfur*	• Histological stain: HE, periodic acid–Schiff staining • Primary keratinocyte cell culture • RT-qPCR • Antibody treatment (to neutralize the IL-36 receptor) • Flow cytometry • Cytotoxicity assay • Epicutaneous infection in mice • Fungal burden	Mice inoculated with *M*. *furfur* show hyperkeratosis and epidermal thickening. *M*. *furfur* triggers a IL17 immune response mediated by the IL-36 receptor through expression of IL17-associated molecules in an epicutaneous mice model, with implications for skin inflammation induced by *Malassezia* infection [21].
In vitro	In vitro cell lines (keratinocytes)	• Easy to manage • Low cost • No ethical implications • Reproducible results • Deep knowledge of cell lines [22]	• Genetically modified cell lines change phenotype and functions • Short observation time • Results cannot be interpolated with in vivo models [22].	*M*. *furfur*	• RT-qPCR • Western blot • ELISA • Cell viability • Negative-stain TEM • Confocal microscopy • IHC	Nanoparticles produced by *M*. *furfur* are internalized into the HaCaT cell line and stimulate IL-6 production [11]
				*M*. *furfur* *M*. *globosa* *M*. *obtusa* *M*. *restricta* *M*. *slooffiae* *M*. *sympodialis*	• ELISA • TEM	Removing the *Malassezia* capsular-like layer triggers a significant increase in the production of IL-6, IL-8, and IL-1a and a decrease in intracellular IL-10 in keratinocytes [23]
				*M*. *pachydermatis*	• Histological stain: Wright’s stain • RT-qPCR • Invasion assay	*M*. *pachydermatis* can invade HaCat cells and triggers a strong cellular response [24]
	Ex vivo model^a^	• Isolated tissue closely mimics natural tissue conditions in the in vivo model [25]	• Technically demanding • Short observation time [25]	*M*. *sympodialis*	• SEM • Confocal microscopy • Histology: HE, periodic acid–Schiff staining • RT-qPCR • Immunoassays • Proteomics	Oleic acid in the skin is associated with direct contact of yeast and keratinocytes, as well as damage to the epidermis. The skin exposed to *Malassezia* in oily conditions expresses IL-18 but not antimicrobial peptide genes [10]
	RHE	• Possibility of incorporating various cell types in combination with keratinocytes • No ethical implications • Reproducible results • Higher degree of standardization [26,27]	• Impairment of barrier function • Lack of equivalent for dermal features (for instance, vasculature, glands, and lipids) • Short observation time • No adaptive immune response [27]	*M*. *furfur* *M*. *sympodialis*	• Light microscopy • Wide-field fluorescence microscopy • SEM • Cytotoxicity assay • RT-qPCR	*M*. *furfur* and *M*.*sympodialis* colonize and form biofilm at the RhE surface [12]

FISH, fluorescence in situ hybridization; GMS, Grocott Gomori methenamine-silver nitrate; HE, hematoxylin–eosin; IHC, immunohistochemistry; MBL, mannose-binding lectin; PDA, pancreatic ductal adenocarcinoma; RhE, reconstructed human epidermis; RT-qPCR, quantitative reverse transcription PCR; SEM, scanning electron microscopy; TEM, transmission electron microscopy.

^a^Explanted organs such as skin explant.

**Fig 1:**
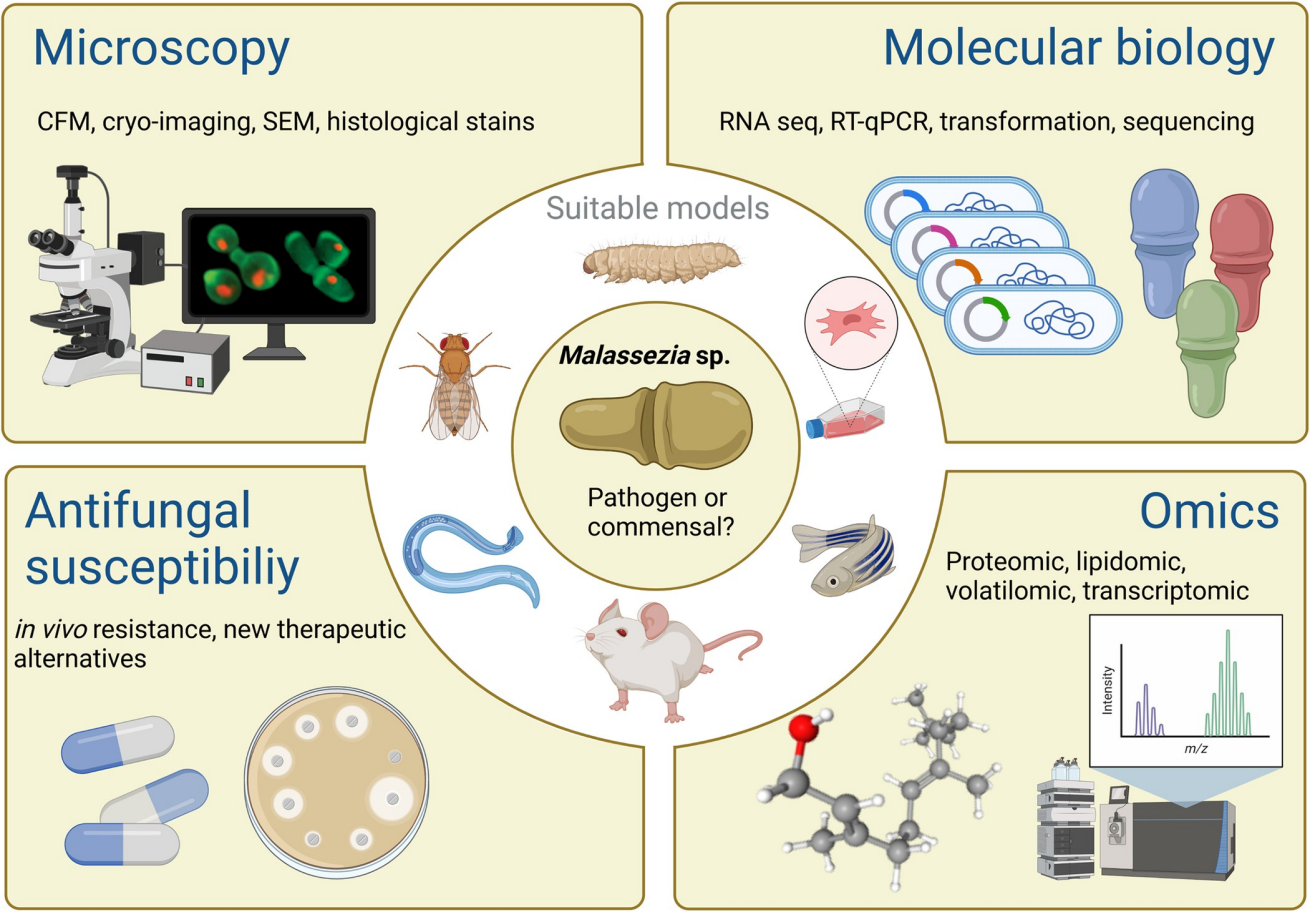
Approaches to study the pathogenicity of *Malassezia* spp. Various in vivo and in vitro models allow the study of infection processes and the evaluation of the host cellular and molecular responses. Multiple methodologies can be used together in experimental models to better understand *Malassezia* metabolism and implications in pathogenicity. The image was designed in Biorender.com. FCM, Fluorescence confocal microscopy; RT-qPCR, quantitative reverse transcription PCR; SEM, scanning electron microscopy.

The combination of various strategies and tools is necessary to further deduce *Malassezia–*host interactions (Fig 1). Omics approaches, such as lipidomics and volatilomics [28,29], may potentially lead to a deeper understanding of the genus by identifying factors associated with its commensal and pathogenic status. Also, considering the emergence of antifungal resistance in this genus, this could facilitate the development of therapeutic strategies that modulate the homeostasis of the yeast without disruption to the host.

Undoubtedly, research on *Malassezia* is still young, underscoring the importance of understanding the lipid requirements of this genus to determine the ideal conditions that allow for the survival of *Malassezia* in the niches that they occupy. In this review, we highlight advances in the field and different strategies that can be used to continue to learn about this genus and its interactions with hosts.

### Beyond what you can see: Is it possible to study *Malassezia* interactions with a model host?

The implementation of microscopy-based techniques is a new field in *Malassezia* research, especially when the goal is to evaluate the interaction between this yeast and its host. Various microscopy-based techniques [30] in combination with other approaches can be implemented to understand whether *Malassezia* can invade the tissue or if the disease is caused by other factors and to document the interaction between *Malassezia* and model hosts.

Microscopy-based techniques can be used to analyze the progression of infection, invasion of cells, colonization, and biofilm formation of *Malassezia* with in vivo or ex vivo models (Table 1). For example, Corzo-León and colleagues used various stains (periodic acid solution, Schiff reagent, counterstain with hematoxylin solution) and microscopic techniques (fluorescent confocal microscopy [FCM] and SEM) to reveal the host–pathogen interaction in oily and non-oily skin in an ex vivo human skin model infected with *M*. *sympodialis* [10]. Used together, these techniques demonstrated a direct interaction between *Malassezia* and keratinocytes and the epidermal damage caused by these fungi [10]. In addition, histological stains have been used to evaluate yeast–host interactions and the cellular response to infection, tissue invasion, and colonization in *G*. *mellonella* and RhE models infected with *Malassezia* [9,12]. Further, FCM has been standardized for costained *Malassezia* and lipid storage organelles (i.e., lipid droplets), which are promoters of pathogenicity and survival during stress conditions in other microorganism models [31].

Microscopy techniques are widely used in other pathogenic fungi to investigate tissue invasion, cell damage, and the host–pathogen interaction. For example, fluorescent imaging methodology was used to visualize the interaction of macrophage cells in zebrafish and *Cryptococcus neoformans* [32]. In this study, Bojarczuk and colleagues established that macrophages could control infection in zebrafish and observed infection progression in detail [32]. Likewise, zebrafish as a model host for fungal infections has also been used in *Candida albicans* because it allows prolonged in vivo imaging of host–pathogen interactions, especially for bloodstream infections [33]. A bronchoscopic fibered confocal fluorescence microscopy (FCFM) technique was implemented and standardized for in vivo visualization and monitoring of *Cryptococcus* and *Aspergillus* infections in murine lungs [34]. This FCFM technique enabled researchers to visualize and describe morphological features of fungal cells during in vivo infection, which provided insight into how the condition progresses [34]. Implementing these techniques to visualize *Malassezia* in the skin could help to better understand how infection develops, the possible physical changes that occur to the yeast in the evolution of disease, and the relationship between the yeast and host macrophages or other cells.

FCM and cryo-imaging techniques have been used to describe the cellular response of the infection of *Aspergillus fumigatus* in *G*. *mellonella* [35]. The cryo-images showed nodule development demonstrating dissemination and melanization indicating tissue invasion [35]. FCM of the nodules confirmed the presence of germinating conidia and hyphae [35]. In combination with other techniques, these approaches could be implemented for *Malassezia* in different host models to answer questions related to physical interactions, dissemination, and invasion that can help to determine whether *Malassezia* is a commensalist or pathogen.

### Focusing on the extraordinary: How could infection models help to determine the unknown function of *Malassezia* genes and the role of gene products in virulence?

In *Malassezia*, variability in virulence has been reported within species and between species [36]. However, it is unclear which genes are involved in this variability and their role in *Malassezia* pathogenesis, homeostasis, or host interactions. At least 44 *Malassezia*-specific gene clusters exist, some likely acquired through horizontal gene transfer. However, most have unknown functions [37]. The linkage between the mating loci in *Malassezia* may be involved in pathogenesis variability [37].

Assessing the function of genes can be challenging due to the biological features of the yeasts belonging to the *Malassezia* genus, including lipid requirements, feasibility of cell contact during transformation processes, growing time, and the cell wall structure [38]. Thus, development of a transformation technique that overcomes these challenging features is much needed for gene function studies.

*A*. *tumefaciens*–mediated transformation provides random insertional mutagenesis and CRISPR/Cas9-mediated targeted gene deletion in *Malassezia* yeasts [38–41]. This tool, in combination with a murine model and macrophages as an ex vivo model, has facilitated the evaluation of the role of *M*. *sympodialis* flavohemoglobin, a protein encoded by a horizontally transferred gene [42]. In both models, flavohemoglobin was not necessary to establish infection in the murine model or for survival inside macrophages [42]. This is the first approach to study *Malassezia* gene function in host–microbe interactions, and more studies are needed.

In addition, *A*. *tumefaciens*–mediated transformation and CRISPR/Cas9–mediated targeted gene deletion can be used to study the mechanism of action of therapeutic strategies, such as calcineurin inhibitors [43]. However, given the large number of genes involved in the *Malassezia* host–microbe interaction and the relative lack of data on these genes, in vivo models are needed. Larvae of *G*. *mellonella* and zebrafish may serve as alternative in vivo systemic infection models. In addition, superficial and systemic infection models in adult zebrafish may provide opportunities to assess virulence and gene function [13,33]. Amorim-Vaz and colleagues evaluated 22 targeted transcription factor mutants of unannotated genes of *C*. *albicans* in *G*. *mellonella*, which demonstrated the reliability of this insect as a fungal infection model with results that correlated with a murine model [44]. Similarly, García-Carnero and colleagues demonstrated the reliability of *G*. *mellonella* larva as a model for *Candida* spp. and mutants with reduced virulence. This study identified predictors of virulence, such as changes in hemocyte circulation, melanization, phenoloxidase, and lactate dehydrogenase activity [45]. A 2-day postfertilization zebrafish larvae systemic infection model has allowed in vivo assessment of the infection process and tissue invasion with *C*. *neoformans*, a species more closely related to *Malassezia* spp. than *C*. *albicans*, with results that correlate with previous observations of the host innate immune response in the murine model [46]. Zebrafish larvae inoculated with *C*. *neoformans* mCherry-expressing deficient mutants and wild-type strains were observed with fluorescence microscopy, demonstrating (i) the mechanism of immune control of the infection and (ii) its ability to survive phagocytosis and invade tissues [46]. These examples demonstrate the feasibility of this experimental design to identify the function of *Malassezia* genes and their role in virulence.

The zebrafish larval infection model is a well-known animal model that is amenable to genetic modification. In addition, this model can be implemented in combination with the *A*. *tumefaciens*–mediated transformation technique to observe in vivo changes in cellular interaction associated with the genes of interest. This may lead to a better understanding of the *Malassezia*–host interaction.

### Comprehending the complex metabolism of *Malassezia*: Is the metabolism of *Malassezia* related to pathogenic processes?

The complex metabolism of *Malassezia* results in the production of different molecules or metabolites that could be involved in the transition from commensal to pathogenic behavior. These molecules include lipids, proteins, and volatile organic compounds (VOCs), knowledge of which could increase the understanding of the pathogenicity of *Malassezia* species, as has been described for other microorganisms [47–49].

The production and assimilation of lipids in yeasts have been studied due to their involvement in membrane composition and their role in regulating cell membrane–associated proteins [50]. The study of lipid metabolism in *Malassezia* is relevant because of the importance of lipids in energy storage, signaling processes, metabolism, and membrane composition [31,47], as well as the fact that *Malassezia* is lipid dependent. Lipid characterization has shown differences between species during the stationary phase, revealing 18 lipid classes and 428 lipidic compounds. These lipids are represented by sterols, triacylglycerols, diglycerides, and fatty acid esters of hydroxy fatty acids. Curiously, the compounds’ concentrations vary between species. For example, the content of cholesteryl ester is lower than other lipid classes (for instance, cholesterol or triacylglycerols) in *M*. *furfur*, atypical *M*. *furfur*, and *M*. *pachydermatis* and undetectable in *M*. *globosa*, *M*. *restricta*, and *M*. *sympodialis* [51]. Moreover, lipidomic and proteomic analyses identified lipid metabolism proteins, most of which are enzymes involved in lipid biosynthesis. The complex lipid metabolism of *Malassezia* may contribute to the genus’s pathogenic processes [51,52]. Other assessments have revealed a connection between the production of lipid mediators in human skin and *Malassezia*, raising questions about the role of these lipids in the establishment of disease [28]. The role of lipids in disease development has been described in a *Saccharomyces cerevisiae* model, in which disturbances in cellular lipid homeostasis resulted in cell death induced by free fatty acid toxicity and lipid peroxidation in the mitochondrial pathways of apoptosis [47]. In *C*. *albicans*, phospholipid pathways enhance virulence; for example, the phosphatidylserine synthase mutant is avirulent in mice and has reduced production of phosphatidylethanolamine, which is thought to be involved in cell wall integrity, mitochondrial function, and filamentous growth [53]. In addition, lipidomic analysis enables the study of lipid composition under specific conditions. For example, lipidomic characterization of *Fusarium oxysporum* isolates infecting *G*. *mellonella* revealed a higher number of phospholipid species with higher unsaturation in clinical versus environmental isolates [54].

Proteins are important in metabolism because they are functional molecules that perform biochemical reactions due to transcriptomic processes [55]. Proteomic analyses can provide information about cell biology, host–pathogen interactions, antimicrobial resistance, biomarker discovery, and identification of anti- and propathogenic cellular responses [55,56]. Few studies have been performed to unravel the role of *Malassezia* proteins in host interactions. One of these involved protein characterization using liquid chromatography with tandem mass spectrometry and found that human skin exposed to *M*. *sympodialis* had increased protein expression by 18%. The proteins reported in that study were mainly related to cornification, antimicrobial immune response, and defense response to fungus [10]. An in vitro study assessed the effect of *M*. *globosa* aspartyl protease 1 (MgSAP1) on *S*. *aureus* biofilm production and found that MgSAP1 could cleave the *S*. *aureus* protein A, which is involved in biofilm production [57], demonstrating a possible protective role of *M*. *globosa* on the skin. Still, it is necessary to evaluate this activity in ex vivo or in vivo models to better understand the effect of this aspartyl protease on a potential pathogen.

Host–pathogen interactions have been assessed via proteomic approaches in other microorganisms. For example, protein expression during *A*. *fumigatus* infection of *G*. *mellonella* revealed increased levels of antimicrobial peptides and proteins that contribute to the innate immune response to fungus in mammals [35]. Similarly, in a *C*. *albicans*–infected G. *mellonella* model, the yeast secreted several proteins related to pathogenesis, oxidative stress, hyphal cell wall formation, and heat shock into the larval hemolymph, which enhances the *C*. *albicans* virulence process [48]. These methodological approaches could be applied to the *Malassezia–*host interaction to identify proteins involved in host interactions, those related to virulence, and those that interact with the host immune response.

Fungal volatiles are gaining relevance because of their involvement in host–pathogen interactions and use as biocontrol alternatives [58]. A recent study detected 61 VOCs in different growth media supplemented with lipids in the *M*. *furfur* exponential and stationary growth phases [49]. This study confirmed chemical differentiation of the VOCs under other conditions. For instance, γ-dodecalactone was identified in the modified Dixon and oleic acid media in both growth phases, but not in palmitic acid or the combination oleic–palmitic acid media. In addition, differences were observed in the production of VOCs according to the growth phase. For instance, dimethyl sulfide increased during the stationary phase, while others, including octane, decreased, suggesting that *Malassezia* VOC production is stimulated by the compounds in the growth media and demonstrating the dynamic metabolism of this yeast [49]. However, no information exists for other species regarding VOC production, function in metabolism, or role in biological interactions. In contrast, the role of VOCs produced by several microorganisms has been described. Gas chromatography with mass spectrometry has demonstrated that VOCs produced by the pathogen *A*. *fumigatus* are toxic to *D*. *melanogaster* on the basis of fungal VOCs interrupting the metamorphic development of the insect. Further, compounds such as 1-octen-3-ol and isopentyl alcohol may increase the pathogenicity of the fungus [58]. In *G*. *mellonella*, a coinfection model demonstrated that sulfur compounds produced by *P*. *aeruginosa* promoted the growth of *A*. *fumigatus* [59]. These findings may help to understand the role of metabolism in virulence during host–pathogen interactions.

### Understanding resistance in *Malassezia*

The study of antifungal resistance in *Malassezia* is of clinical interest because *Malassezia* spp. have been identified as the etiologic agents of bloodstream infections and are associated with severe systemic disease [60–62]. Considering the recurrence of *Malassezia* skin disease and the need for long-term antifungal treatment, precautions are needed to prevent the emergence of resistance to various classes of antifungal agents [62,63]. However, due to the nutritional requirements of this genus, it has been difficult to standardize interlaboratory methodologies for in vivo and in vitro analyses [64]. As such, several groups have modified the in vitro Clinical and Laboratory Standards Institute M27-A3 and European Committee on Antimicrobial Susceptibility Testing protocols by adjusting temperature and supplementing culture medium with different lipids [65]. Nonetheless, it is important to note that there is no common agreement in a standard methodology as there is for pathogenic yeasts such as *Candida* and *Cryptococcus*, and the lack of clinical cutoff values hinders the determination of resistance [65].

Recent studies have reported isolates from different *Malassezia* spp. with high minimum inhibitory concentrations (MICs) against some azoles, such as fluconazole, voriconazole, and ketoconazole [62,66,67]. Given the growing evidence of the emergence of azole resistance in *Malassezia*, there is increasing interest in understanding the mechanisms behind this phenomenon. Hence, various approaches have been used to understand resistance in *Malassezia*, including (i) synergism studies in which combinations of azoles and efflux pump inhibitors like haloperidol, pro-methazine, and cyclosporine A reduced azole MICs compared to treatment without inhibitors in *M*. *furfur* and *M*. *pachydermatis* [68]; (ii) genomic studies in which ketoconazole resistance in a *M*. *pachydermatis* isolate was attributed to duplication of genes encoding ERG4 and ERG11 [69] and findings that duplication of ATM1 and ERG11 could explain resistance to this azole in *M*. *restricta* [67]; and (iii) gene expression and RNA-seq analyses that demonstrated that efflux pumps such as PDR5 [67] and PDR10 [61] are up-regulated in resistant isolates of *M*. *restricta* and *M*. *furfur*, respectively, exposed to azoles (ketoconazole and clotrimazole). The latter was also confirmed via CRISPR-Cas9 deletion of PDR10. These results demonstrated this efflux pump provides azole resistance in *M*. *furfur* and is not only attributed to CYP51 mutations as reported previously [61].

Although the use of antifungal agent susceptibility profiling is increasing *Malassezia*, much remains unknown about the in vivo course of antifungal treatment. Mammalian models are the standard to evaluate the efficacy and pharmacokinetics of novel and traditional antimicrobial agents [70]. Regardless, there is a growing interest in using other models of infection, such as *G*. *mellonella*, considering ethical issues with mammalian models [71,72]. The *G*. *mellonella* invertebrate model has been used in different infection models to study pharmacokinetics due to similar responses found in humans [70,72]. Although *Malassezia* infections have been successfully established in murine models and *G*. *mellonella*, no antifungal activity assays have been performed [9,73]. Thus, in vivo models and methodologies that allow the evaluation of novel antifungal or alternative therapeutic treatments are needed [64].

Considering that antifungal susceptibility tests have been performed on other yeasts and filamentous fungi in a wide variety of experimental models, background studies support standardization of a model for *Malassezia*. For instance, 2 antifungal compounds have been evaluated in a coculture of *Trichophyton rubrum* and keratinocytes in which the expression of genes related to therapeutic targets and resistance mechanisms was determined by quantitative RT-PCR [74]. In addition, *G*. *mellonella* has been used to verify an in vitro–in vivo correlation of the combined effect of antifungal treatments on Mucorales growth [75]. As another example, a zebrafish model was proposed for antifungal compound screening in *C*. *albicans* infection [76]. Likewise, murine models have been used to determine the activity of new molecules, such as occidiofungin and T-2307, against *C*. *albicans* and *C*. *neoformans*, respectively, with a correlation between in vitro and in vivo results [77,78].

New therapeutic candidates and alternatives have been proposed for *Malassezia*-associated infections. For instance, L-lysine [79], AMP, essential oils, and plant extracts [80] have been proposed as alternatives to traditional antifungals therapies, but further research is needed to study their effectiveness in vivo [79]. It is crucial to standardize models to evaluate the efficacy, safety, and pharmacokinetics of traditional and alternative therapies and to corroborate whether there is a correlation between in vivo and in vitro antifungal efficacy.

## Conclusions

Considering the efficacy of current techniques applied to in vivo and in vitro models to understand host–pathogen relationships and aspects related to metabolism in other fungal species, it would be worthwhile to introduce these tools to the study of *Malassezia*. These models can be used to inform the application of strategies to identify the role of different molecules in host interactions. Even identification of the pathways by which these metabolites are produced could clarify the behavior of *Malassezia*. Such studies will make it possible to propose alternative therapeutic targets to control infections caused by these yeasts or improve diagnostic techniques.

## References

[1] Saheb Kashaf S, Proctor DM, Deming C, Saary P, Hölzer M, Taylor ME, et al. Integrating cultivation and metagenomics for a multi-kingdom view of skin microbiome diversity and functions. Nat Microbiol. 2022;7:169–179. doi: 10.1038/s41564-021-01011-w 34952941 PMC8732310

[2] Vijaya Chandra SH, Srinivas R, Dawson TL Jr, Common JE. Cutaneous *Malassezia*: commensal, pathogen, or protector? Front Cell Infect Microbiol. 2021;10:869. doi: 10.3389/fcimb.2020.614446 33575223 PMC7870721

[3] Abdillah A, Ranque S. Chronic Diseases Associated with *Malassezia* Yeast. J Fungi. 2021;7:855. doi: 10.3390/jof7100855 34682276 PMC8540640

[4] Ianiri G, Heitman J. Approaches for genetic discoveries in the skin commensal and pathogenic *Malassezia* yeasts. Front Cell Infect Microbiol. 2020;393. doi: 10.3389/fcimb.2020.00393 32850491 PMC7426719

[5] Sparber F, De Gregorio C, Steckholzer S, Ferreira FM, Dolowschiak T, Ruchti F, et al. The skin commensal yeast *Malassezia* triggers a type 17 response that coordinates anti-fungal immunity and exacerbates skin inflammation. Cell Host Microbe. 2019;25:389–403. doi: 10.1016/j.chom.2019.02.002 30870621

[6] Limon JJ, Tang J, Li D, Wolf AJ, Michelsen KS, Funari V, et al. *Malassezia* is associated with Crohn’s disease and exacerbates colitis in mouse models. Cell Host Microbe. 2019;25:377–388. doi: 10.1016/j.chom.2019.01.007 30850233 PMC6417942

[7] Aykut B, Pushalkar S, Chen R, Li Q, Abengozar R, Kim JI, et al. The fungal mycobiome promotes pancreatic oncogenesis via activation of MBL. Nature. 2019;574:264–267. doi: 10.1038/s41586-019-1608-2 31578522 PMC6858566

[8] Kong G, Lê Ca KA, Hannan AJ. Alterations in the Gut Fungal Community in a Mouse Model of Huntington’s Disease. Microbiol Spectr. 2022:e02192–e02121. 10.1128/spectrum.02192-21 PMC9045163 35262396

[9] Torres M, Pinzón EN, Rey FM, Martinez H, Parra Giraldo CM, Celis Ramírez AM. *Galleria mellonella* as a novelty in vivo model of host-pathogen interaction for *Malassezia furfur* CBS 1878 and *Malassezia pachydermatis* CBS 1879. Front Cell Infect Microbiol. 2020;10:199. doi: 10.3389/fcimb.2020.00199 32432057 PMC7214729

[10] Corzo-León DE, MacCallum DM, Munro CA. Host responses in an *ex vivo* human skin model challenged with *Malassezia sympodialis*. Front Cell Infect Microbiol. 2021;862. 10.3389/fcimb.2020.561382 PMC7859105 33552997

[11] Zhang YJ, Han Y, Sun YZ, Jiang HH, Liu M, Qi RQ, et al. Extracellular vesicles derived from *Malassezia furfur* stimulate IL-6 production in keratinocytes as demonstrated in *in vitro* and *in vivo* models. J Dermatol Sci. 2019;93:168–175. doi: 10.1016/j.jdermsci.2019.03.001 30904352

[12] Pedrosa AF, Lisboa C, Branco J, Pellevoisin C, Miranda IM, Rodrigues AG. *Malassezia* interaction with a reconstructed human epidermis: Keratinocyte immune response. Mycoses. 2019;62:932–936. doi: 10.1111/myc.12965 31278884

[13] Torres M, De Cock H, Celis AM. In vitro or in vivo models, the next frontier for unraveling interactions between *Malassezia* spp. and hosts. How much do we know?. J Fungi. 2020;6:155. doi: 10.3390/jof6030155 32872112 PMC7558575

[14] Madende M, Albertyn J, Sebolai O, Poh CH. *Caenorhabditis elegans* as a model animal for investigating fungal pathogenesis. Med Microbiol Immunol. 2020;209:1–13. doi: 10.1007/s00430-019-00635-4 31555911

[15] Harnish JM, Link N, Yamamoto S. *Drosophila* as a model for infectious diseases. Int J Mol Sci. 2021;22:2724. doi: 10.3390/ijms22052724 33800390 PMC7962867

[16] Merkel S, Heidrich D, Danilevicz CK, Scroferneker ML, Zanette RA. *Drosophila melanogaster* as a model for the study of *Malassezia pachydermatis* infections. Vet Microbiol. 2018;224:31–33. doi: 10.1016/j.vetmic.2018.08.021 30269787

[17] Brilhante RSN, da Rocha MG, de Melo GM, de Oliveira JS, dos Santos G, Acosta JD, et al. *Malassezia pachydermatis* from animals: Planktonic and biofilm antifungal susceptibility and its virulence arsenal. Vet Microbiol. 2018;220:47–52. doi: 10.1016/j.vetmic.2018.05.003 29885800

[18] Hohl TM. Overview of vertebrate animal models of fungal infection. J Immunol Methods. 2014;410:100–112. doi: 10.1016/j.jim.2014.03.022 24709390 PMC4163114

[19] Schlemmer KB, Jesus FP, Loreto ÉS, Tondolo JS, Ledur PC, Dallabrida A, et al. An experimental murine model of otitis and dermatitis caused by *Malassezia pachydermatis*. Mycoses. 2018;61:954–958. doi: 10.1111/myc.12839 30106183

[20] Lee K, Zhang I, Kyman S, Kask O, Cope EK. Co-infection of *Malassezia sympodialis* With Bacterial Pathobionts *Pseudomonas aeruginosa* or *Staphylococcus aureus* Leads to Distinct Sinonasal Inflammatory Responses in a Murine Acute Sinusitis Model. Front Cell Infect Microbiol. 2020;10:472. doi: 10.3389/fcimb.2020.00472 33014894 PMC7498577

[21] Miyachi H, Wakabayashi S, Sugihira T, Aoyama R, Saijo S, Koguchi-Yoshioka H, et al. Keratinocyte IL-36 Receptor/MyD88 Signaling Mediates *Malassezia*-Induced IL-17–Dependent Skin Inflammation. J Infect Dis. 2021;223:1753–1765. doi: 10.1093/infdis/jiab194 33837391

[22] Kaur G, Dufour JM. Cell lines: Valuable tools or useless artifacts: Valuable tools or useless artifacts. Spermatogenesis. 2012;2:1–5. doi: 10.4161/spmg.19885 22553484 PMC3341241

[23] Thomas DS, Ingham E, Bojar RA, Holland KT. *In vitro* modulation of human keratinocyte pro- and anti-inflammatory cytokine production by the capsule of *Malassezia* species. FEMS Immunol Med Microbiol. 2008;54:203–214. doi: 10.1111/j.1574-695X.2008.00468.x 18752620

[24] Buommino E, De Filippis A, Parisi A, Nizza S, Martano M, Iovane G, et al. Innate immune response in human keratinocytes infected by a feline isolate of *Malassezia pachydermatis*. Vet Microbiol. 2013;163:90–96. doi: 10.1016/j.vetmic.2012.12.001 23273837

[25] Fröhlich E, Salar-Behzadi S. Toxicological assessment of inhaled nanoparticles: role of *in vivo*, *ex vivo*, *in vitro*, and *in silico* studies. Int J Mol Sci. 2014;15:4795–4822. doi: 10.3390/ijms15034795 24646916 PMC3975425

[26] Setijanti HB, Rusmawati E, Fitria R, Erlina T, Adriany R, Murtiningsih. Development the technique for the preparation and characterization of reconstructed human epidermis (RHE). Alternatives to Animal Testing. Singapore: Springer Singapore; 2019. p. 20–32.

[27] Suhail S, Sardashti N, Jaiswal D, Rudraiah S, Misra M, Kumbar SG. Engineered skin tissue equivalents for product evaluation and therapeutic applications. Biotechnol J. 2019;14:1900022. doi: 10.1002/biot.201900022 30977574 PMC6615970

[28] Ambaw YA, Pagac MP, Irudayaswamy AS, Raida M, Bendt AK, Torta FT, et al. Host/*Malassezia* Interaction: A Quantitative, Non-Invasive Method Profiling Oxylipin Production Associates Human Skin Eicosanoids with *Malassezia*. Metabolites. 2021;11:700. doi: 10.3390/metabo11100700 34677414 PMC8538739

[29] Rios-Navarro A, Gonzalez M, Carazzone C, Celis Ramírez AM. Learning about microbial language: possible interactions mediated by microbial volatile organic compounds (VOCs) and relevance to understanding *Malassezia* spp. metabolism. Metabolomics. 2021;17:1–1. 33825999 10.1007/s11306-021-01786-3 PMC8026438

[30] Spatz M, Richard ML. Overview of the Potential Role of *Malassezia* in Gut Health and Disease. Front Cell Infect Microbiol. 2020;10:1–11. 32528901 10.3389/fcimb.2020.00201 PMC7265801

[31] Mantilla MJ, Cabrera Díaz CE, Ariza-Aranguren G, de Cock H, Helms JB, Restrepo S, et al. Back to the Basics: Two Approaches for the Identification and Extraction of Lipid Droplets from *Malassezia pachydermatis* CBS1879 and *Malassezia globosa* CBS7966. Curr Protoc. 2021;1:e122. doi: 10.1002/cpz1.122 33950584

[32] Bojarczuk A, Miller KA, Hotham R, Lewis A, Ogryzko NV, Kamuyango AA, et al. *Cryptococcus neoformans* Intracellular Proliferation and Capsule Size Determines Early Macrophage Control of Infection. Sci Rep. 2016;6:21489. doi: 10.1038/srep21489 26887656 PMC4757829

[33] Rosowski EE, Knox BP, Archambault LS, Huttenlocher A, Keller NP, Wheeler RT, et al. The zebrafish as a model host for invasive fungal infections. J Fungi. 2018;4:136. doi: 10.3390/jof4040136 30551557 PMC6308935

[34] Vanherp L, Poelmans J, Hillen A, Govaerts K, Belderbos S, Buelens T, et al. Bronchoscopic fibered confocal fluorescence microscopy for longitudinal *in vivo* assessment of pulmonary fungal infections in free-breathing mice. Sci Rep. 2018;8:3009. doi: 10.1038/s41598-018-20545-4 29445211 PMC5813038

[35] Sheehan G, Clarke G, Kavanagh K. Characterization of the cellular and proteomic response of *Galleria mellonella* larvae to the development of invasive aspergillosis. BMC Microbiol. 2018;18:63. doi: 10.1186/s12866-018-1208-6 29954319 PMC6025711

[36] Rhimi W, Theelen B, Boekhout T, Otranto D, Cafarchia C. *Malassezia* spp. yeasts of emerging concern in fungemia. Front Cell Infect Microbiol. 2020;10:370. doi: 10.3389/fcimb.2020.00370 32850475 PMC7399178

[37] Wu G, Zhao H, Li C, Rajapakse MP, Wong WC, Xu J, et al. Genus-wide comparative genomics of *Malassezia* delineates its phylogeny, physiology, and niche adaptation on human skin. PLoS Genet. 2015;11:e1005614. doi: 10.1371/journal.pgen.1005614 26539826 PMC4634964

[38] Ianiri G, Averette AF, Kingsbury JM, Heitman J, Idnurm A. Gene function analysis in the ubiquitous human commensal and pathogen *Malassezia* genus. MBio. 2016;7:e01853–e01816. doi: 10.1128/mBio.01853-16 27899504 PMC5137500

[39] Ianiri G, Dagotto G, Sun S, Heitman J. Advancing functional genetics through *Agrobacterium*-mediated insertional mutagenesis and CRISPR/Cas9 in the commensal and pathogenic yeast *Malassezia*. Genetics. 2019;212:1163–1179. doi: 10.1534/genetics.119.302329 31243056 PMC6707463

[40] Celis AM, Vos AM, Triana S, Medina CA, Escobar N, Restrepo S, et al. Highly efficient transformation system for *Malassezia furfur* and *Malassezia pachydermatis* using *Agrobacterium tumefaciens*-mediated transformation. J Microbiol Methods. 2017;134:1–6. doi: 10.1016/j.mimet.2017.01.001 28064034

[41] Goh JP, Ianiri G, Heitman J, Dawson TL Jr. Expression of a *Malassezia* codon optimized mCherry fluorescent protein in a bicistronic vector. Front Cell Infect Microbiol. 2020;367. doi: 10.3389/fcimb.2020.00367 32793513 PMC7387403

[42] Ianiri G, Coelho MA, Ruchti F, Sparber F, McMahon TJ, Fu C, et al. HGT in the human and skin commensal *Malassezia*: A bacterially derived flavohemoglobin is required for NO resistance and host interaction. Proc Natl Acad Sci. 2020;117 (27):15884–15894. doi: 10.1073/pnas.2003473117 32576698 PMC7354939

[43] Ianiri G, Applen Clancey S, Lee SC, Heitman J. FKBP12-dependent inhibition of calcineurin mediates immunosuppressive antifungal drug action in *Malassezia*. MBio. 2017;8:e01752–e01717. doi: 10.1128/mBio.01752-17 29066552 PMC5654937

[44] Amorim-Vaz S, Delarze E, Ischer F, Sanglard D, Coste AT. Examining the virulence of *Candida albicans* transcription factor mutants using *Galleria mellonella* and mouse infection models. Front Microbiol. 2015;6:367. doi: 10.3389/fmicb.2015.00367 25999923 PMC4419840

[45] García-Carnero LC, Clavijo-Giraldo DM, Gómez-Gaviria M, Lozoya-Pérez NE, Tamez-Castrellón AK, López-Ramírez LA, et al. Early Virulence Predictors during the *Candida* Species–*Galleria mellonella* Interaction. J Fungi. 2020;6:152. doi: 10.3390/jof6030152 32867152 PMC7559698

[46] Tenor JL, Oehlers SH, Yang JL, Tobin DM, Perfect JR. Live imaging of host-parasite interactions in a zebrafish infection model reveals cryptococcal determinants of virulence and central nervous system invasion. MBio. 2015;6:e01425–e01415. doi: 10.1128/mBio.01425-15 26419880 PMC4611042

[47] Eisenberg T, Büttner S. Lipids and cell death in yeast. FEMS Yeast Res. 2014;14:179–197. doi: 10.1111/1567-1364.12105 24119111 PMC4255311

[48] Sheehan G, Kavanagh K. Proteomic analysis of the responses of *Candida albicans* during infection of *Galleria mellonella* larvae. J Fungi. 2019;5:1–12. doi: 10.3390/jof5010007 30641883 PMC6463115

[49] Gonzalez M, Celis AM, Guevara-Suarez MI, Molina J, Carazzone C. Yeast smell like what they eat: Analysis of Volatile Organic Compounds of *Malassezia furfur* in Growth Media Supplemented with Different Lipids. Molecules. 2019;24:419. doi: 10.3390/molecules24030419 30678374 PMC6384859

[50] Singh P. Budding yeast: An ideal backdrop for in vivo lipid biochemistry. Front Cell Dev Biol. 2017;4:1–8. doi: 10.3389/fcell.2016.00156 28119915 PMC5222803

[51] Celis AM, Amézquita A, Cardona JEC, Matiz-Cerón LF, Andrade-Martínez JS, Triana S, et al. Analysis of *Malassezia* Lipidome Disclosed Differences Among the Species and Reveals Presence of Unusual Yeast Lipids. Front Cell Infect Microbiol. 2020;10:1–15. 32760678 10.3389/fcimb.2020.00338 PMC7374198

[52] Triana S, de Cock H, Ohm RA, Danies G, Wösten HAB, Restrepo S, et al. Lipid metabolic versatility in *Malassezia* spp. yeasts studied through metabolic modeling. Front Microbiol. 2017;8:1772. doi: 10.3389/fmicb.2017.01772 28959251 PMC5603697

[53] Chen YL, Montedonico AE, Kauffman S, Dunlap JR, Menn FM, Reynolds TB. Phosphatidylserine synthase and phosphatidylserine decarboxylase are essential for cell wall integrity and virulence in *Candida albicans*. Mol Microbiol. 2010;75:1112–1132. doi: 10.1111/j.1365-2958.2009.07018.x 20132453

[54] Sepulveda-rivera J, Jimenez P, Jaramillo CP, Marin PA. Biological Activity of Lipids Extracted from Two Isolates of *Fusarium oxysporum* (Environmental and Clinical) in *Galleria mellonella*. 2020;7:1–7.

[55] Saleh S, Staes A, Deborggraeve S, Gevaert K. Targeted Proteomics for Studying Pathogenic Bacteria. Proteomics. 2019;19:1–10. doi: 10.1002/pmic.201800435 31241236

[56] Jean Beltran PM, Federspiel JD, Sheng X, Cristea IM. Proteomics and integrative omic approaches for understanding host–pathogen interactions and infectious diseases. Mol Syst Biol. 2017;13:922. doi: 10.15252/msb.20167062 28348067 PMC5371729

[57] Li H, Goh BN, Teh WK, Jiang Z, Goh JP, Goh A, et al. Skin commensal *Malassezia globosa* secreted protease attenuates *Staphylococcus aureus* biofilm formation. J Invest Dermatol. 2018;138:1137–1145. doi: 10.1016/j.jid.2017.11.034 29246799

[58] Almaliki HS, Angela A, Goraya NJ, Yin G, Bennett JW. Volatile Organic Compounds Produced by Human Pathogenic Fungi Are Toxic to *Drosophila melanogaster*. Front Fungal Biol. 2021;1:1–11. 10.3389/ffunb.2020.629510 PMC10512272 37743879

[59] Scott J, Sueiro-Olivares M, Ahmed W, Heddergott C, Zhao C, Thomas R, et al. *Pseudomonas aeruginosa*-Derived Volatile Sulfur Compounds Promote *Distal Aspergillus* fumigatus Growth and a Synergistic Pathogen-Pathogen Interaction That Increases Pathogenicity in Coinfection. Front Microbiol. 2019;10:1–15. 31649650 10.3389/fmicb.2019.02311 PMC6794476

[60] Iatta R, Cafarchia C, Cuna T, Montagna O, Laforgia N, Gentile O, et al. Bloodstream infections by *Malassezia* and *Candida* species in critical care patients. Med Mycol. 2014;52:264–269. doi: 10.1093/mmy/myt004 24576998

[61] Leong C, Chan J, Lee S, Lam Y, Goh J, Ianiri G, et al. Azole Resistance Mechanisms in Pathogenic *Malassezia furfur*. Antimicrob Agents Chemother. 2021;65:e01975–e01920. 10.1128/AAC.01975-20 PMC8092866 33619053

[62] Pedrosa A, Lisboa C, Faria-Ramos I, Silva R, Ricardo E, Teixeira-Santos R, et al. Epidemiology and susceptibility profile to classic antifungals and over-the-counter products of *Malassezia* clinical isolates from a Portuguese University Hospital: a prospective study. J Med Microbiol. 2019;68:778–784. doi: 10.1099/jmm.0.000966 30907722

[63] Ashbee H. Update on the genus *Malassezia*. Med Mycol. 2007;45:287–303. doi: 10.1080/13693780701191373 17510854

[64] Saunte D, Gaitanis G, Hay R. *Malassezia*-Associated Skin Diseases, the Use of Diagnostics and Treatment. Front Cell Infect Microbiol. 2020;10:112. doi: 10.3389/fcimb.2020.00112 32266163 PMC7098993

[65] Peano A, Pasquetti M, Tizzani P, Chiavassa E, Guillot J, Johnson E. Methodological Issues in Antifungal Susceptibility Testing of *Malassezia pachydermatis*. J Fungi. 2017;3:37. doi: 10.3390/jof3030037 29371554 PMC5715951

[66] Cafarchia C, Iatta R, Immediato D, Puttilli M, Otranto D. Azole susceptibility of *Malassezia pachydermatis* and *Malassezia furfur* and tentative epidemiological cut-off values. Med Mycol. 2015;53:743–748. doi: 10.1093/mmy/myv049 26162472

[67] Park M, Cho Y, Lee Y, Jung W. Genomic Multiplication and Drug Efflux Influence Ketoconazole Resistance in *Malassezia restricta*. Front Cell Infect Microbiol. 2020;10:191. doi: 10.3389/fcimb.2020.00191 32426297 PMC7203472

[68] Iatta R, Puttilli M, Immediato D, Otranto D, Cafarchia C. The role of drug efflux pumps in *Malassezia pachydermatis* and *Malassezia furfur* defence against azoles. Mycoses. 2017;60:178–182. doi: 10.1111/myc.12577 27774659

[69] Kim M, Cho Y, Park M, Choi Y, Hwang S, Jung W. Genomic Tandem Quadruplication is Associated with Ketoconazole Resistance in *Malassezia pachydermatis*. J Microbiol Biotechnol. 2018;28:1937–1945. doi: 10.4014/jmb.1810.10019 30562885

[70] Hill L, Veli N, Coote P. Evaluation of *Galleria mellonella* larvae for measuring the efficacy and pharmacokinetics of antibiotic therapies against *Pseudomonas aeruginosa* infection. Int J Antimicrob Agents. 2014;43:254–261. doi: 10.1016/j.ijantimicag.2013.11.001 24361354

[71] Desbois A, Coote P. Utility of Greater Wax Moth Larva (*Galleria mellonella*) for Evaluating the Toxicity and Efficacy of New Antimicrobial Agents. Adv Appl Microbiol. 2012;78:25–53. doi: 10.1016/B978-0-12-394805-2.00002-6 22305092

[72] Jemel S, Guillot J, Kallel K, Botterel F, Dannaoui E. *Galleria mellonella* for the Evaluation of Antifungal Efficacy against Medically Important Fungi, a Narrative Review. Microorganisms. 2020;8:390. 10.3390/microorganisms8030390 PMC7142887 32168839

[73] Sparber F, LeibundGut-Landmann S. Infecting Mice with *Malassezia* spp. to Study the Fungus-Host Interaction. J Vis Exp. 2019;153:e60175. 10.3791/60175 31762452

[74] Komoto T, Bitencourt T, Silva G, Beleboni R, Marins M, Fachin A. Gene Expression Response of *Trichophyton rubrum* during Coculture on Keratinocytes Exposed to Antifungal Agents. Evid Based Complement Alternat Med. 2015;2015:1–7. doi: 10.1155/2015/180535 26257814 PMC4516844

[75] Macedo D, Leonardelli F, Dudiuk C, Vitale R, Del Valle E, Giusiano G, et al. In Vitro and *In Vivo* Evaluation of Voriconazole-Containing Antifungal Combinations against Mucorales Using a *Galleria mellonella* Model of Mucormycosis. J Fungi. 2019;5:5. doi: 10.3390/jof5010005 30626083 PMC6462937

[76] Kulatunga D, Dananjaya S, Nikapitiya C, Kim C, Lee J, De Zoysa M. *Candida albicans* Infection Model in Zebrafish (*Danio rerio*) for Screening Anticandidal Drugs. Mycopathologia. 2019;184:559–572. doi: 10.1007/s11046-019-00378-z 31473909

[77] Mitsuyama J, Nomura N, Hashimoto K, Yamada E, Nishikawa H, Kaeriyama M, et al. *In Vitro* and *In Vivo* Antifungal Activities of T-2307, a Novel Arylamidine. Antimicrob Agents Chemother. 2008;52:1318–1324. doi: 10.1128/AAC.01159-07 18227186 PMC2292552

[78] Ravichandran A, Geng M, Hull K, Li J, Romo D, Lu S, et al. A Novel Actin Binding Drug with *In Vivo* Efficacy. Antimicrob Agents Chemother. 2019;63:e01585–e01518. doi: 10.1128/AAC.01585-18 30323040 PMC6325233

[79] Sastoque A, Triana S, Ehemann K, Suarez L, Restrepo S, Wösten H, et al. New Therapeutic Candidates for the Treatment of *Malassezia pachydermatis* -Associated Infections. Sci Rep. 2020;10. 32184419 10.1038/s41598-020-61729-1 PMC7078309

[80] Rhimi W, Theelen B, Boekhout T, Aneke C, Otranto D, Cafarchia C. Conventional therapy and new antifungal drugs against *Malassezia* infections. Med Mycol. 2020;59:215–234. 10.1093/mmy/myaa087 33099634

